# Overexpressing *GRE3* in *Saccharomyces cerevisiae* enables high ethanol production from different lignocellulose hydrolysates

**DOI:** 10.3389/fmicb.2022.1085114

**Published:** 2022-12-19

**Authors:** Haijie Wang, Limin Cao, Qi Li, Nalin N. Wijayawardene, Jian Zhao, Min Cheng, Qi-Rui Li, Xiaobin Li, Itthayakorn Promputtha, Ying-Qian Kang

**Affiliations:** ^1^Key Laboratory of Medical Microbiology and Parasitology & Key Laboratory of Environmental Pollution Monitoring and Disease Control, Ministry of Education, School of Basic Medical Sciences, Guizhou Medical University, Guiyang, Guizhou, China; ^2^Beijing Key Laboratory of Plant Gene Resources and Biotechnology for Carbon Reduction and Environmental Improvement, College of Life Sciences, Capital Normal University, Beijing, China; ^3^Center for Yunnan Plateau Biological Resources Protection and Utilization, College of Biological Resource and Food Engineering, Qujing Normal University, Qujing, China; ^4^Section of Genetics, Institute for Research and Development in Health and Social Care, Battaramulla, Sri Lanka; ^5^National Institute of Fundamental Studies, Kandy, Sri Lanka; ^6^State key Laboratory of Microbial Technology, Shandong University, Qingdao, China; ^7^Department of Hospital Infection Management, Affiliated Hospital of Guizhou Medical University, Guiyang, Guizhou, China; ^8^Chishui Riverside Jiangi-Flavour Baijiu Research Center, Guizhou Sunveen Liquor Co., Ltd, Guiyang, China; ^9^Department of Biology, Faculty of Science, Chiang Mai University, Chiang Mai, Thailand; ^10^Environmental Science Research Center, Faculty of Science, Chiang Mai University, Chiang Mai, Thailand

**Keywords:** ethanol, *GRE3*, lignocellulosic hydrolysates, *Saccharomyces cerevisiae*, xylose

## Abstract

The efficiently renewable bioethanol can help to alleviate energy crisis and environmental pollution. Genetically modified strains for efficient use of xylose and developing lignocellulosic hydrolysates play an essential role in facilitating cellulosic ethanol production. Here we present a promising strain GRE3^OE^ via *GRE3* overexpressed in a previously reported *Saccharomyces cerevisiae* strain WXY70. A comprehensive evaluation of the fermentation level of GRE3^OE^ in alkaline-distilled sweet sorghum bagasse, sorghum straw and xylose mother liquor hydrolysate. Under simulated corn stover hydrolysate, GRE3^OE^ produced 53.39 g/L ethanol within 48 h. GRE3^OE^ produced about 0.498 g/g total sugar in sorghum straw hydrolysate solution. Moreover, GRE3^OE^ consumed more xylose than WXY70 in the high-concentration xylose mother liquor. Taken together, GRE3^OE^ could be a candidate strain for industrial ethanol development, which is due to its remarkable fermentation efficiency during different lignocellulosic hydrolysates.

## Introduction

With the higher demand for fossil fuel, the atmospheric CO_2_ level is rising. Thus, biofuels have become increasingly popular as sustainable and renewable alternative energy sources (e.g., bioethanol; Sheehan, 2009). Bioethanol is environmentally friendly and abundant. Hence, used for biofuel, which is an alternative to fossil fuel due to biodegradability and reduced toxicity (Baghel et al., 2015; Vasić et al., 2021). Corn and sugarcane are mainly used as inputs in the production of bioethanol in Brazil and the United States. As well as sustainability issues, there are limitations to the using of food resources (Greene et al., 2004; Farrell et al., 2006). It is necessary to increase ethanol production for green environmental and economic sustainability. Renewable cellulosic ethanol is gaining more attention due to its low cost and ability to reduce environmental pollution.

The two most abundant components in lignocellulose feedstock hydrolysates are glucose and xylose (Jeffries, 1985). In co-fermentations of glucose and xylose, industrially robust microbes have not been available. It has been found that *Saccharomyces cerevisiae* produces ethanol with a high yield and productivity, making it a good candidate for bioethanol production (Lee et al., 2016). *Saccharomyces cerevisiae* has been reported with high capacity for converting glucose into ethanol (Piskur et al., 2006). The artificial introduction of xylose metabolism genes (including XR/XDH pathway and XI pathway) into *Saccharomyces cerevisiae* results in efficiently mixed sugar co-fermentation for high ethanol production (Lee et al., 2021). In view of this, the development of high bioethanol-producing yeast strains from cellulose hydrolysates is a timely needed and a vital task.

However, wild-type *Saccharomyces cerevisiae* is incapable of utilizing xylose during the fermentation process. Therefore, a variety of strain development procedures have been used to create *Saccharomyces cerevisiae* that can quickly and effectively ferment xylose. Biomass is efficiently converted into ethanol, xylose utilization is more important to industries. Recently, genetically modified *Saccharomyces cerevisiae* have attracted great attention for their ability to efficiently utilize xylose (Lee et al., 2021). *Saccharomyces cerevisiae* utilized xylose through a xylose reductase/xylitol dehydrogenase (XR/XDH) pathway and a xylose isomerase (XI) pathway (Cao et al., 2014; Bae et al., 2021). Compared with strains using XI pathway, XR/XDH pathway strains consume xylose more efficiently and produce more ethanol production (Karhumaa et al., 2007). Previously, the XR/XDH expression strains were successfully constructed *in-vitro* and showed a trend towards increased ethanol production (Zhang et al., 2019; Sun and Jin, 2021). Through this pathway that xylitol can be converted to xylulose by the NADPH-dependent enzyme xylose reductase (XR), encoded by *XYL1*. By NAD^+^-dependent xylitol dehydrogenase (XDH) encoded by *XYL2*, xylulose is converted (Zha et al., 2012). The *GRE3* gene encodes a non-specific aldose reductase containing NADPH as a co-factor (Romaní et al., 2015). The *GRE3* genes of *Candida albicans* and *Saccharomyces cerevisiae*, and the *XYL1* gene of *Scheffersomyces stipitis*, are highly related sequences encoding predicted xylose reductase activity (Harcus et al., 2013). The expression levels of *GRE3*, *XYL2* and *XYL3* could be optimized to further resolve the xylose redox imbalance (Kim et al., 2017). We speculate that *GRE3* has similar potential to *XYL1* and *XYL2* in terms of xylose utilization and plays a critical role in xylose metabolism.

There is still much more endeavor for improvement in ethanol production due to the high concentration of mixed sugars and inhibitors of various microorganisms produced by industrial physicochemical pretreatment of lignocellulosic feedstocks. In this study, a gene closely associated with ethanol metabolism by introducing *GRE3*, into XR/XDH utilized yeast strain to improve the biofuel production from cellulose hydrolysates and to provide a potential industrial strain for the development of efficient cellulosic ethanol. In different hydrolysates obtained by different pretreatment methods, modification yeast strains were evaluated for fermentation efficiency. In this study, the overexpression of *GRE3* can improve ethanol production in different hydrolysates, using the parental strain WXY70 as a reference. We finally confirm that GRE3^OE^ is a promising strain in industrial applications.

## Materials and methods

### The construction of yeast strains and plasmids

Previously evolved strain CE7 that nos. 15,567 stored in China General Microbiological Culture Collection Center, the XR-XDH-XK pathway was introduced to obtain strain WXY70 by Zhang et al. (2019). An amplified copy of the *GRE3* gene was linked to a linearized fragment T1 from plasmid pT1-0 (L1-P*_PGK1_*-T*_PGI_*-L2) using the Golden Gate assembly method to produce plasmid pT1-1 (Zhang et al., 2019). By linearizing pT1-1 with PCR following *DpnI* digestion, we obtained the fragments L1-P*_PGK1_*-*GRE3*-T*_PGI_*-L2. In addition, plasmids pT5-0 and pT3-0 contained linearized fragments CAT8up-AbA-L1 and L2-CAT8down. WXY70 was produced by co-transforming these three fragments. In the confirmed strain, P*_PGK1_*-*GRE3*-T*_PGI_* is integrated at the CAT8 site and drives overexpression of *GRE3*, which is known as GRE3^OE^.

### Simulated corn stover hydrolysate fermentation analysis

Yeast cells were grown in normal YPD (Difco, United States) medium shaking at 200 rpm for 24 h, then centrifuged for 5 min and washed twice with sterile water before collection. Inoculated yeast was inoculated into 250 ml shake flasks with 100 ml of simulated corn stover hydrolysate (80 g/l glucose, 40 g/l xylose, and 3 g/l acetate) at an OD600 value of 1 (Zhang et al., 2019). To reduce air exchange, fermentation was performed under anaerobic conditions at 30°C, 200 rpm, and three layers of parafilm. The samples were retrieved by syringe needle at every 12 h time interval for HPLC analysis. Three replicates were performed for each experiment.

### Alkaline distillation of sweet sorghum bagasse hydrolysate fermentation

A solid-state fermentation produced sweet sorghum with a diameter of 1 to 2 mm and a length of 3 to 50 mm. The alkaline-distilled process is carried out by completely mixing approximately 4 kg of sweet sorghum bagasse with 10% (w/w DM) NaOH concentrate solution, which is then loaded into distillation stripper designed and manufactured by Tsinghua University. Hydrolysis method according to the described by Yu et al. (2014). A solid-to-liquid ratio of 1: 5 is achieved after hydrolysis, the insoluble solids removed by centrifugation and then supplemented 2 g/l KH_2_PO_4_, 2 g/l (NH_4_)_2_SO_4_, 1 g/l MgSO_4_ and 10 g/l yeast extract for fermentation. Initial fermentation cell density was OD_600_ = 1.0. Three replicates were performed for each experiment.

### Sorghum straw hydrolysate fermentation

The pretreatment of sorghum straw hydrolysate solution was based on Wu et al. (2021). A collection of sorghum straw (SS) in the suburbs of Lianyungang (China) and processed through a 40 mesh sieve grinder. An enclosed conical flask with a liquid–solid ratio of 10: 1 (wt: wt) was used. SS was pretreated in dilute NaOH (0.75 wt%) solution and then placed in an autoclave at 121°C for 1 h (Wu et al., 2021). An exact ratio of choline chloride (ChCl) and lactic acid (LAC) was mixed. To reduce evaporation, 60°C was applied to the mixture in a conical flask with a stopper and stirred continuously (160 rpm) until a clear solvent was obtained. An oil bath at 150°C was used to mix 75.0 g of ChCl: LAC with 5.0 g of SS in a three-necked flask. Pretreated SS was also obtained by washing and dehydrating the mixture. A triplicate sample of the hydrolysis was prepared, while other hydrolysate samples were stored at 4°C for future use.

### Analytical procedures for yeast fermentation processes

Determination of substrate concentrations in samples including mixed sugars and metabolites containing ethanol and xylitol using a 1,260 HPLC column HPX-87H (Bio-Rad, United States) at 30°C and a relative index of refraction detector at 50°C. The mobile phase used in the HPLC system was 5 mM H_2_SO_4_ flowing at a rate of 0.4 ml/min (Zhang et al., 2019). Xylose mother liquor with a sugar content of 120 g/l xylose (Jin et al., 2019). An analytical grade is used for all chemicals.

## Result and discussion

### Construction of GRE3^OE^ strains and fermentation analysis

**Figure 1:**
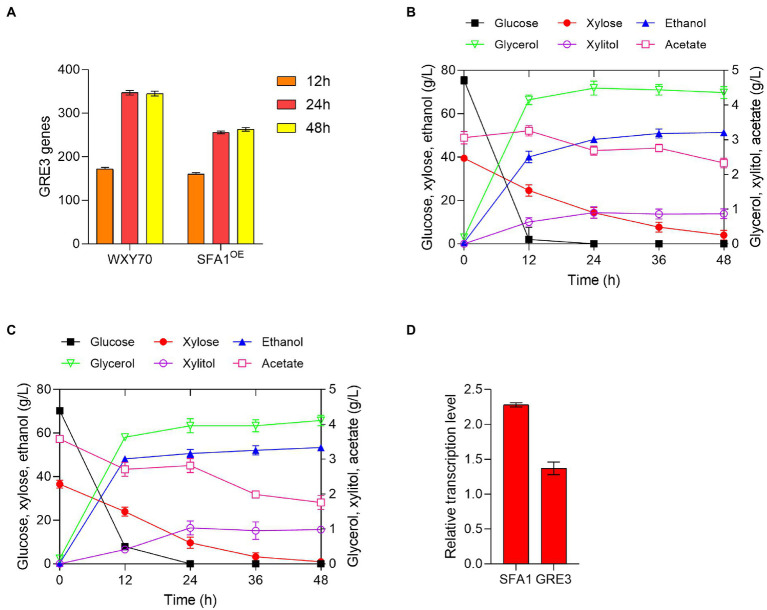
Analysis of transcript levels of engineered strains and fermentation in simulated corn stover hydrolysate solution. **(A)** Amount of *GRE3* genes in strains WXY70 and SFA1^OE^. Fermentation profiles of the parental strain WXY70 **(B)**, GRE3^OE^ **(C)** in simulated corn stover hydrolysate within 48 h. **(D)** The transcriptional level analysis of SFA1^OE^ and GRE3^OE^ strains overexpressed genes.

Our previously reported strain SFA1^OE^ by expressing *SFA1* gradually increased ethanol production in different lignocellulosic hydrolysates (Zhu et al., 2020). As shown in Figure 1A, we found differences in *GRE3* expressed genes displayed upregulated transcription trend according to our transcriptome analysis in strains WXY70 and SFA1^OE^ during the fermentation process, based on the efficient fermentation performance of SFA1^OE^ and the excellent ethanol production, it is reasonable to hypothesize that *GRE3* gene expression is positively correlated with ethanol production and could further improve fermentation in industrial settings.

In this study, GRE3^OE^ was constructed by expressing *GRE3* in the parental strain WXY70 and fermented in the simulated corn stover hydrolysate (Table 1). At 12 h, WXY70 and GRE3^OE^ consumed all glucose, and gradually increased ethanol production. During fermentation, the remaining xylose contents of WXY70 and GRE3^OE^ were 4.03 and 0.96 g/l, respectively (Figures 1B,C). At 48 h, WXY70 and GRE3^OE^ produced 51.37 and 53.39 g/l of the maximum ethanol concentrations, or ethanol yield of 0.447 and 0.498 g/g total sugars, approximately 87.65 and 97.65% of the theoretical value, proving that GRE3^OE^ performed better during fermentation to produce ethanol and improved xylose metabolic capacities. In addition, WXY70 consumed less acetate (0.73 g/l) than GRE3^OE^ (1.82 g/l). A mixture of glucose and xylose resulted in significant improvements in ethanol production by GRE3^OE^. The results suggest that applying the XR/XDH to engineered yeast strains during fermentation can be a promising strategy. In contrast, deletion of the *GRE3* gene also yielded 67.8% of the theoretical value for ethanol in mixed sugar fermentation (Tanino et al., 2010). Additionally, *Saccharomyces cerevisiae* strain SR8 fermented xylose excellently, with an ethanol yield of 0.359 g/g total sugars (Shin et al., 2019). Above results suggest that the *GRE3* is efficient in regulating the consume of sugars and has a positive effect on the efficient of glucose and xylose consumption as well as the rate of ethanol production.

**Table 1:** Comparison of fermentation profile in simulated corn stover hydrolysate fermentation between WXY70 and GRE3^OE^ of the 48 h.

Parameters	WXY70	GRE3^OE^
Initial glucose concentration (g/L)	75.45 ± 1.74	70.29 ± 1.16
Initial xylose concentration (g/L)	39.47 ± 1.40	36.55 ± 1.81
Maximum ethanol concentration (g/L)	51.37 ± 1.25	53.39 ± 1.02
Ethanol yield (g/g)	0.447 ± 0.012	0.498 ± 0.011
Ethanol productivity (g/L·h)	1.07 ± 0.03	1.11 ± 0.04
Xylitol production (g/L)	0.87 ± 0.15	0.98 ± 0.10
Xylitol productivity (g/L·h)	0.02 ± 0.00	0.02 ± 0.00
Glycerol production (g/L)	4.36 ± 0.12	4.11 ± 0.15
Glycerol productivity (g/L·h)	0.09 ± 0.00	0.08 ± 0.00

To better validate these results, we examined the transcript levels of SFA1^OE^ and GRE3^OE^ as shown in Figure 1D. Interestingly, the expression levels of *SFA1* and *GRE3* were increased, which were 2.28 and 1.37 times of WXY70. Overexpression of *GRE3* may further overcome the redox imbalance in xylose utilization, resulting in increased ethanol production. A transcriptomic analysis of yeast strains according to its foldchange revealed positive correlations between previously reported key genes *SFA1*, *CCW12* and *SPT2* expressions and the ethanol production improvements (Li et al., 2022). Based on the previously reported excellent ethanol production performance of the SFA1^OE^ strain (Zhu et al., 2020), thus, it is speculated that high ethanol yield in GRE3^OE^ is closely related to the changes in their expression levels. In summary, we speculated that expression of *GRE3* resulted in upregulation of *CDC19* which encoding enzymes involved in pyruvate to ethanol pathway, yielding high ethanol production. The above results motivated us to measure the fermentability of GRE3^OE^ in a different of hydrolysates to further evaluation of the fermentation profile of the GRE3^OE^ strain.

### Evaluation of the fermentation capacity of GRE3^OE^ in alkaline-distilled sweet sorghum bagasse

In addition to providing plenty of soluble sugars, sweet sorghum bagasse has a high lignocellulosic content, making it a potential source of energy crop for the production of bioethanol. During the integrated fermentation process, soluble mixture sugars from released sweet sorghum hydrolysate can be fermented through a proposed Advanced Solid-state Fermentation technology to produce efficient 1.5 generation bioethanol production (Li et al., 2013), an alkali-distillation process combines ethanol distillation and alkali pretreatment in order to convert sweet sorghum bagasse’s lignocellulose into ethanol (Li et al., 2013). This advanced technology increases the efficiency of sweet sorghum bagasse utilization through a green, low consumption process.

**Figure 2:**
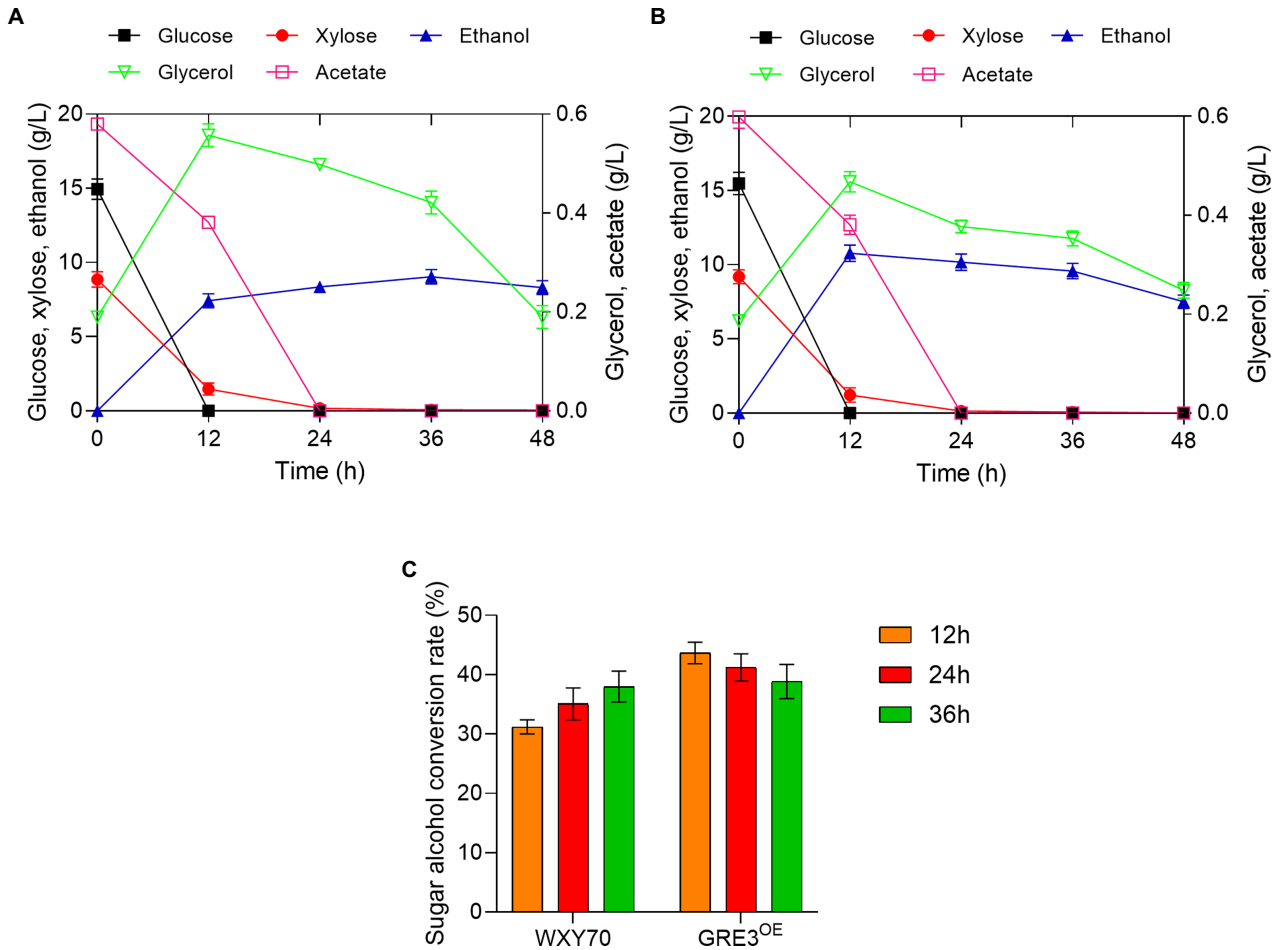
Profiles of strain WXY70 and GRE3^OE^ during fermentation and growth in alkaline-distilled sweet sorghum bagasse hydrolysate solution. Fermentation and growth profile of WXY70 **(A)** and GRE3^OE^ **(B)** within 48 h. **(C)** The sugar alcohol conversion rate of WXY70 and GRE3^OE^ at 12 h, 24 h, and 36 h.

Considering that sweet sorghum bagasse with high mixed sugar content, the feasibility of producing cellulosic ethanol from alkaline-distilled sweet sorghum bagasse with GRE3^OE^ was evaluated (Table 2). Within fermentation process of 12 h, both strains WXY70 and GRE3^OE^ consumed almost all the glucose. The xylose was consumed at 8.79 g/l and 9.19 g/l for WXY70 and GRE3^OE^, respectively. GRE3^OE^ has better xylose consumption capacity. At 12 h, WXY70 produced 7.43 g/l ethanol, with an ethanol yield of 0.310 g/g total sugars, or 60.70% of the theoretical value (Figure 2A), GRE3^OE^ produced 10.77 g/l ethanol, with an ethanol yield of 0.431 g/g total sugars, or 84.47% of the theoretical value (Figure 2B). GRE3^OE^ produced the maximum ethanol concentration earlier than WXY70. GRE3^OE^ consumed more sugar and thus produced more ethanol than WXY70 at the same time. As shown in Figures 2A,B, acetate as an inhibitor can inhibit the growth of yeast cells, GRE3^OE^ consumed slightly acetate (0.60 g/l) than WXY70 (0.58 g/l), indicating that GRE3^OE^ contributes in the consumption inhibitor, allowing the strain to produce ethanol during the fermentation more effectively. Based on these results, we also observed that sugar alcohol conversion of GRE3^OE^ was higher than WXY70. At 12 h, the sugar alcohol conversion of WXY70 and GRE3^OE^ were 31.20 and 43.66% (Figure 2C). Within 36 h, the sugar alcohol conversion rate of GRE3^OE^ was higher than that of WXY70, suggesting that GRE3^OE^ is more efficient at converting sugar to ethanol in a short period of time. The increased sugar alcohol conversion of GRE3^OE^ may be due to a further improvement in the redox imbalance in xylose metabolism. The above results show that GRE3^OE^ had much better fermentation performance than WXY70.

**Table 2:** Comparison of fermentation profile in alkaline-distilled sweet sorghum bagasse fermentation between WXY70 and GRE3^OE^ of the 48 h.

Parameters	WXY70	GRE3^OE^
Initial glucose concentration (g/L)	14.95 ± 1.21	15.47 ± 0.96
Initial xylose concentration (g/L)	8.85 ± 0.90	9.19 ± 0.82
Maximum ethanol concentration (g/L)	9.04 ± 0.90	10.77 ± 0.93
Ethanol production (g/g)	0.377 ± 0.037	0.431 ± 0.040
Ethanol productivity (g/L·h)	0.25 ± 0.03	0.90 ± 0.06
Glycerol production (g/L)	0.19 ± 0.04	0.25 ± 0.01
Glycerol productivity (g/L·h)	0.004 ± 0.001	0.005 ± 0.001

We previously reported an efficient engineered strain SFA1^OE^ (Zhu et al., 2020), a yeast strain with efficient fermentation performance in alkaline-distilled sweet sorghum bagasse, we consider GRE3^OE^ to be similar to SFA1^OE^. Thus, we evaluated the fermentation performance of GRE3^OE^ under alkaline-distilled sweet sorghum bagasse. As expected, GRE3^OE^ produced more cellulosic ethanol from alkaline-distilled sweet sorghum bagasse hydrolysate than control strain WXY70. Contrary to previous reports that strain *Zymomonas mobilis* TSH-01 produced 5.65 g/l ethanol, or 0.161 g/g total sugar under the same condition of alkaline-distilled sweet sorghum bagasse (Zhu et al., 2020). Above this overexpression of *GRE3* genes in *Saccharomyces cerevisiae* improves the fermentation performance. The alkaline distillation of lignocellulosic materials is considered as a cost-effective method of producing bioethanol from 1.5 generations of biomass. For the production of bioethanol, GRE3^OE^ strain ensures high efficacy and complete utilization of sweet sorghum bagasse.

### Evaluation of the fermentation capacity of GRE3^OE^ in sorghum straw hydrolysate solution

**Figure 3:**
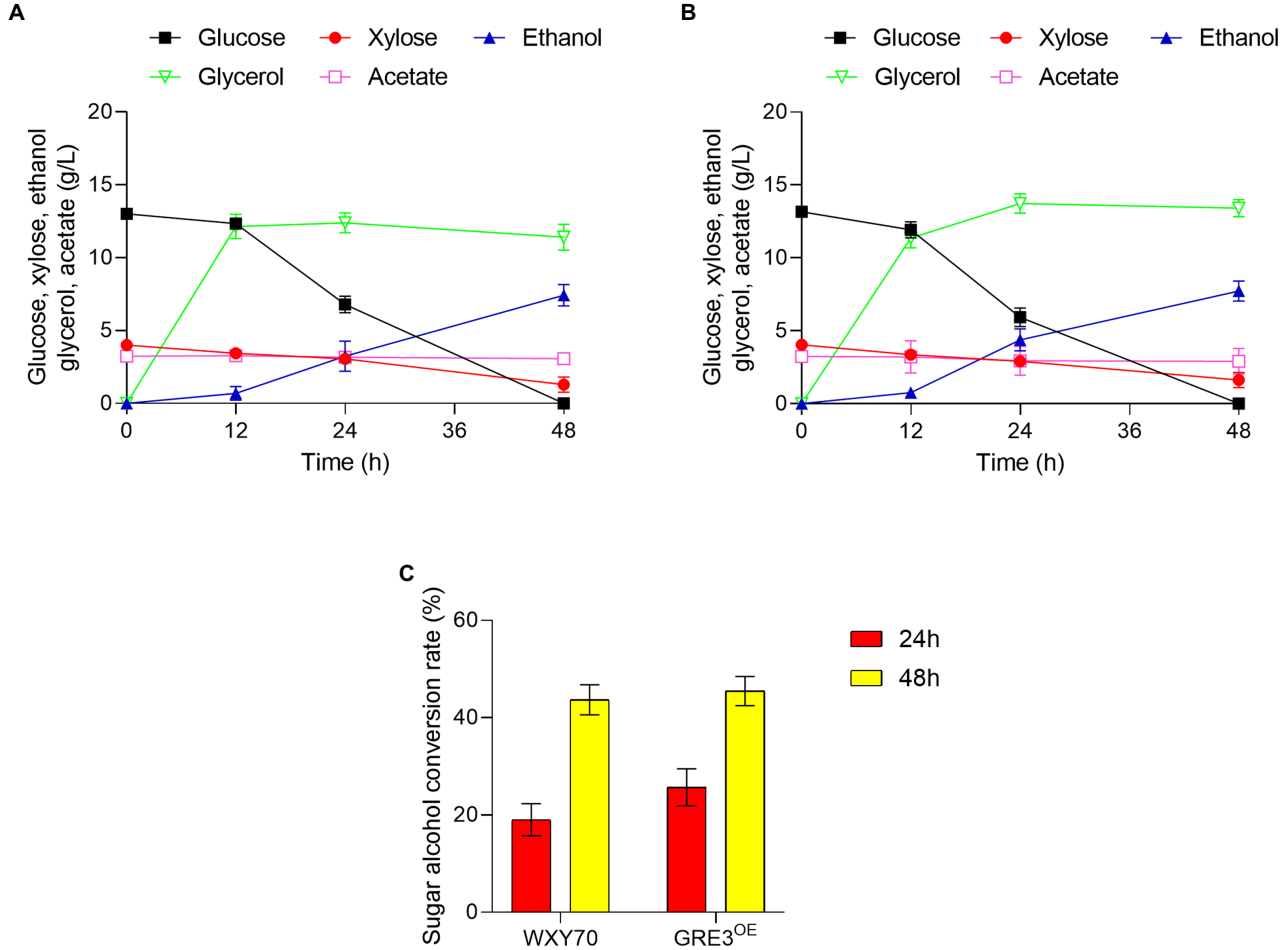
The growth and fermentation characteristics of strain WXY70 and GRE3^OE^ in sorghum straw hydrolysate solution. Growth and fermentation characteristics of WXY70 **(A)** and GRE3^OE^ **(B)** within 48 h. **(C)** The sugar alcohol conversion rate of WXY70 and GRE3^OE^ at 24 h and 48 h.

Straw from sorghum is a renewable feedstock that can be used to produce biofuels and biobased chemicals (Matsakas et al., 2014). We evaluated the fermentability of WXY70 and GRE3^OE^ in sorghum straw hydrolysate solution (Table 3). Within fermentation process of 48 h, both strains WXY70 and GRE3^OE^ consumed almost all the glucose, the control strain WXY70 produced 7.43 g/l ethanol production, with an ethanol yield of 0.437 g/g total sugars, or 85.70% of the theoretical maximum (Figure 3A). Meanwhile GRE3^OE^ produced 7.73 g/l ethanol, with an ethanol yield of 0.455 g/g total sugars, or 89.16% of the theoretical maximum (Figure 3B). Our fermentation results showed that GRE3^OE^ produced slightly ethanol production than WXY70 in sorghum straw hydrolysate solution.

**Table 3:** Comparison of fermentation profile in sorghum straw hydrolysate fermentation between WXY70 and GRE3^OE^ of the 48 h.

Parameters	WXY70	GRE3^OE^
Initial glucose concentration (g/L)	13.01 ± 1.31	13.15 ± 1.09
Initial xylose concentration (g/L)	4.00 ± 1.25	4.04 ± 1.18
Maximum ethanol concentration (g/L)	7.43 ± 1.27	7.73 ± 1.20
Ethanol yield (g/g)	0.437 ± 0.064	0.455 ± 0.041
Ethanol productivity (g/L·h)	0.15 ± 0.03	0.16 ± 0.03
Xylitol production (g/L)	0.00 ± 0.00	0.00 ± 0.00
Xylitol productivity (g/L·h)	0.00 ± 0.00	0.00 ± 0.00
Glycerol production (g/L)	11.40 ± 1.14	13.42 ± 1.02
Glycerol productivity (g/L·h)	0.24 ± 0.02	0.27 ± 0.01

However, WXY70 produced 11.40 g/l glycerol while GRE3^OE^ produced 13.42 g/l at 48 h. We observed that the GRE3^OE^ strain produced more glycerol than WXY70. The close contact due to *GRE3* and *GPD1*, a gene that controls glycerol synthesis (Aguilera and Prieto, 2004). Research has been carried out to improve ethanol production by overexpressing *GRE3* instead of *XYL1* in *Saccharomyces cerevisiae* (Kim et al., 2013), and we reasonably hypothesize that *GRE3* is correlated with *XYL1* and both of them are aldose reductases. Overexpression of *XYL1*, regulated by the strong promoter of *PGK1*, increased the XR activity of the strains in the study, and an increase in glycerol production in strains with high XR activity was also observed (Jeppsson et al., 2003). We speculate that *GRE3* overexpression increased XR activity and led to upregulation of *GPD1* thus increasing ethanol and glycerol production. Acetate is the main inhibitor in industrial fermentation solutions. At the same time, WXY70 consumed 0.16 g/l acetate while GRE3^OE^ consumed 0.34 g/l.

As shown in Figure 3C, the sugar alcohol conversion of WXY70 and GRE3^OE^ was 19.04 and 25.67% at 24 h. At 48 h the sugar alcohol conversion of WXY70 and GRE3^OE^ was 43.68 and 45.46%. This fermentation result showed GRE3^OE^ has higher sugar alcohol conversion than WXY70. Thus, our fermentation results suggested *GRE3* enhances the sugar utilization efficiency in sorghum straw hydrolysates solution resulted in more ethanol production.

### Evaluation of the fermentation capacity of GRE3^OE^ in xylose mother liquor

**Figure 4:**
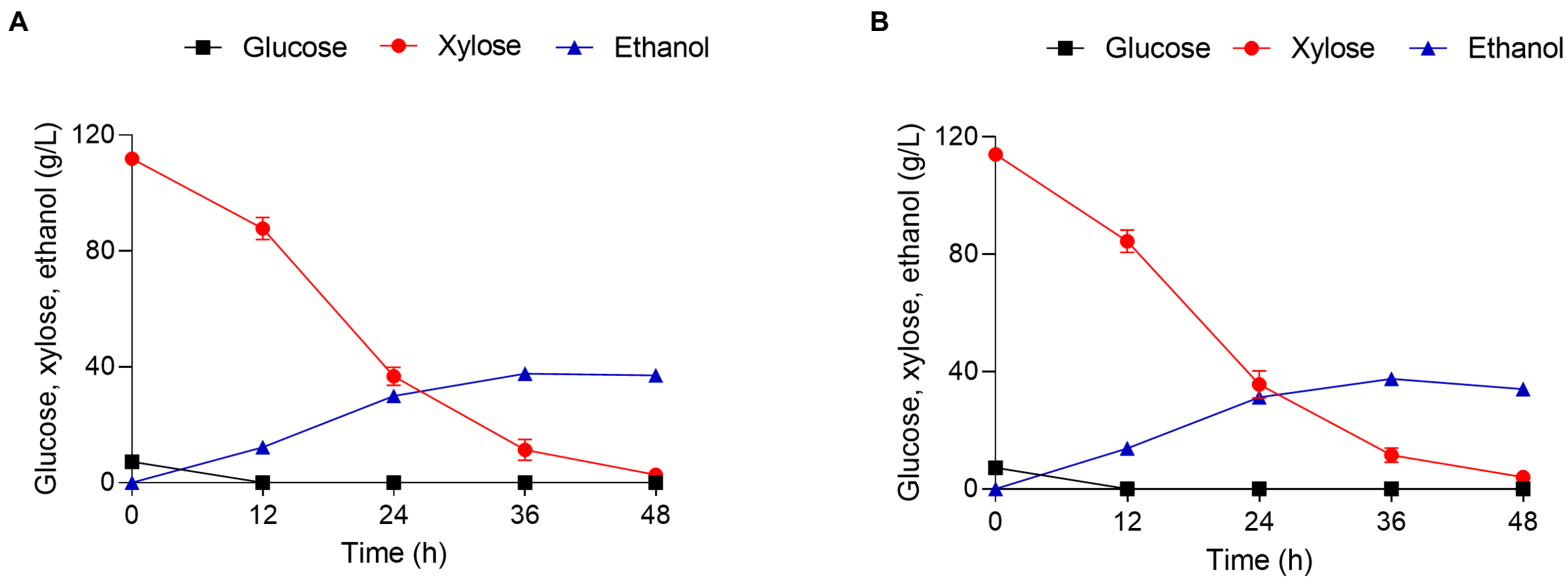
Xylose mother liquor hydrolysate fermentation and growth characteristics of strains WXY70 and GRE3^OE^. Growth and fermentation characteristics of WXY70 **(A)** and GRE3^OE^ **(B)** within 48 h.

Functional sugar alcohols can be produced from the xylose mother liquor (XML) generated during xylitol production (Jin et al., 2019). However, there is a great deal of difficulty in directly separating or reusing this xylose (Wang et al., 2016; Liu et al., 2018). We have previously determined the relative fermentation profiles of the highly efficient engineered strains SPT2^OE^ and 8G-3 in 120 g/l xylose and they have good fermentation performance (Li et al., 2022). Thus, the relative fermentation profiles of WXY70 and GRE3^OE^ under the same experimental conditions were determined (Table 4). As shown in Figures 4A,B, both strains consumed all the glucose at 24 h. During the fermentation process, the remaining xylose content of WXY70 and GRE3^OE^ strains was 4.04 g/l and 2.80 g/l, suggesting that GRE3^OE^ consumed more xylose to produce high ethanol production. At 36 h, the control strain WXY70 produced 37.57 g/l ethanol, with an ethanol yield of 0.310 g/g total sugar or 60.71% of the theoretical maximum (Figure 4A). In contrast, GRE3^OE^ produced 37.66 g/l ethanol, with an ethanol yield of 0.315 g/g total sugar, or 62.00% of the theoretical maximum (Figure 4B). The above results indicated that GRE3^OE^ strain accelerated xylose metabolism and improved xylose utilization efficiency to produce more ethanol production.

**Table 4:** Comparison of fermentation profile in xylose mother liquor hydrolysate fermentation between WXY70 and GRE3^OE^ of the 48 h.

Parameter	WXY70	GRE3^OE^
Initial glucose concentration (g/L)	7.26 ± 0.03	7.27 ± 0.01
Initial xylose concentration (g/L)	114.08 ± 1.23	111.82 ± 1.26
Maximum ethanol concentration (g/L)	37.57 ± 1.22	37.66 ± 1.31
Ethanol yield (g/g)	0.310 ± 0.001	0.315 ± 0.001
Ethanol productivity (g/L·h)	1.04 ± 0.01	1.05 ± 0.02

In addition, *GRE3*, *XYL2*, and *XYL3* overexpressing strains exhibited decent XR and XDH activities (Kim et al., 2017). *GRE3* has a potential function in xylose utilization and plays a critical role in xylose metabolism through XR/XDH pathway. An additional copy of XR resulted in a 1.7-fold increase in xylose consumption and a 55% decrease in xylitol yield in a previous study (Jeppsson et al., 2003). It is possible to improve xylose utilization by balancing the XR activity (Karhumaa et al., 2006). Meanwhile, engineered strains use the *PGK1* promoter to increase XR activity, resulting in assimilating and metabolizing xylose faster (Zha et al., 2012). Our fermentation results in this study also using a stronger promoter *PGK1* to express *GRE3* further increase xylose consumption as well as increase ethanol production. Although previous studies reported that engineered strain with deletion of the *GRE3* could increase ethanol production (Träff et al., 2001; Tanino et al., 2010). Their strains are based on the XI/XK pathway, while our strains are based on the XR/XDH pathway. We used different xylose metabolic pathways and our fermentation results showed that due to the XR/XDH pathway GRE3^OE^ strains could also perform well in ethanol production.

In this study, these fermentation results indicated our metabolic engineering modification strategies are rational. The engineered industrial strain demonstrated efficacious xylose utilization. Besides, we observed that the engineered *Saccharomyces cerevisiae* overexpressing *GRE3* exhibited better xylose fermentation and ethanol production than the control strain, which is also consistent with previous studies (Träff-Bjerre et al., 2003; Kim et al., 2013). Taken together, GRE3^OE^ exhibited superior fermentation properties in comparison with that of WXY70 in complex hydrolysate environments, resulting in its potential industrial ethanol application.

## Conclusion

The target strain GRE3^OE^ was obtained by metabolic and evolutionary engineering. Alkaline-distilled sweet sorghum bagasse, sorghum straw, and xylose mother liquor was used to evaluate GRE3^OE^ contribution to cellulosic ethanol production. GRE3^OE^ outperformed the control strain in simulated corn stover hydrolysate within 48 h, achieving an over 53 g/l of ethanol. GRE3^OE^ produced ethanol yield of 0.498 g/g total sugars within 48 h in sorghum straw hydrolysate solution. These results indicate that GRE3^OE^ outperformed control strain in alkaline-distilled sweet sorghum bagasse, sorghum straw and xylose mother liquor hydrolysate solution. As a result of this study, a new pathway for efficient bioethanol production has opened up and a potential industrial strain has been identified. In the future, we can positively focus on the expression of the *GRE3* to contribute ethanol production. Based on our obtained results in this study, GRE3^OE^ has a high glycerol production, we can achieve transformation by combining *GPD1* and *GRE3* expressing. The further research work promotes the conversion of glycerol into ethanol by expressing glycerol dehydrogenase gene and pull more carbon towards ethanol rather than the by-products, which increasing the yield and rate of ethanol.

## Data availability statement

The original contributions presented in the study are included in the article/supplementary material; further inquiries can be directed to the corresponding author.

## Author contributions

HW: data curation and writing original draft. LC: conceptualization and writing review and editing. QL: formal analysis and data curation. NW, XL, IP, and Q-RL: writing and review. JZ and MC: data curation. Y-QK: funding acquisition and writing and review. All authors contributed to the article and approved the submitted version.

## Funding

This work was supported by the 111 Project (D20009); National Natural Science Foundation of China (NSFC; no. 32060034 and 22278273); State Key Laboratory of Microbial Technology Open Projects Fund (Project NO. M2022-09); Guizhou Scientific Plan Project ([2020]4Y220); Talent Base Project of Guizhou Province, China [RCJD2018-22]; Guizhou Provincial Academician Workstation of Microbiology and Health (No. [2020]4004); High-level Innovation Talent Project of Guizhou Province (GCC [2022]036–1); and Major Science and Technology Projects of China Tobacco [No.110202101048 (LS-08)].

## Conflict of interest

XL was employed by company Guizhou Sunveen Liquor Co., Ltd.

The remaining authors declare that the research was conducted in the absence of any commercial or financial relationships that could be construed as a potential conflict of interest.

## Publisher’s note

All claims expressed in this article are solely those of the authors and do not necessarily represent those of their affiliated organizations, or those of the publisher, the editors and the reviewers. Any product that may be evaluated in this article, or claim that may be made by its manufacturer, is not guaranteed or endorsed by the publisher.
